# Evaluation of a Diet Quality Index Based on the Probability of Adequate Nutrient Intake (PANDiet) Using National French and US Dietary Surveys

**DOI:** 10.1371/journal.pone.0042155

**Published:** 2012-08-03

**Authors:** Eric O. Verger, François Mariotti, Bridget A. Holmes, Damien Paineau, Jean-François Huneau

**Affiliations:** 1 AgroParisTech, CRNH-IdF, UMR914 Nutrition Physiology and Ingestive Behavior, Paris, France; 2 INRA, CRNH-IdF, UMR914 Nutrition Physiology and Ingestive Behavior, Paris, France; 3 Danone Research, Nutrition Department, Palaiseau, France; University of Ottawa, Canada

## Abstract

**Background:**

Existing diet quality indices often show theoretical and methodological limitations, especially with regard to validation.

**Objective:**

To develop a diet quality index based on the probability of adequate nutrient intake (PANDiet) and evaluate its validity using data from French and US populations.

**Material and Methods:**

The PANDiet is composed of adequacy probabilities for 24 nutrients grouped into two sub-scores. The relationship between the PANDiet score and energy intake were investigated. We evaluated the construct validity of the index by comparing scores for population sub-groups with ‘a priori’ differences in diet quality, according to smoking status, energy density, food intakes, plasma folate and carotenoid concentrations. French and US implementations of the PANDiet were developed and evaluated using national nutritional recommendations and dietary surveys.

**Results:**

The PANDiet was not correlated with energy for the French implementation (r = −0.02, *P*>0.05) and correlated at a low level for the US implementation (r = −0.11, *P*<0.0001). In both implementations, a higher PANDiet score (i.e. a better diet quality) was associated with not smoking, having a lower-energy-dense diet, consuming higher amounts of fruits, vegetables, fish, milk and other dairy products and lower amounts of cheese, pizza, eggs, meat and processed meat, and having higher plasma folate and carotenoid concentrations after controlling for appropriate factors (all *P*<0.05, carotenoid data for US not available).

**Conclusions:**

The PANDiet provides a single score that measures the adequacy of nutrient intake and reflects diet quality. This index is adaptable for use in different countries and relevant at the individual and population levels.

## Introduction

Nutritional epidemiology typically involves the analysis of associations between a specific nutrient, food or food category, and health-related outcomes. Such an approach fails to consider the complexity of the diet as a whole, which includes multiple correlations between foods and nutrients. Dietary patterns are complementary to classical analyses because they can tackle the diet complexity using a holistic approach [1]. There are two main approaches for characterizing dietary patterns in a population. The first approach, known as ‘a posteriori’, uses data-driven techniques such as principal component analysis and cluster analysis [2], [3]. The second approach, known as ‘a priori’, defines dietary patterns based on current nutrition knowledge, mainly expressed as food or nutrient based dietary guidelines [4]–[6]. The overall adherence or proximity to these dietary patterns is used to build indices of diet quality. The majority of existing indices are based on the traditional Mediterranean diet or national food-based dietary guidelines.

One practical drawback of the food-based dietary guidelines approach is that indices can rarely be applied to populations with different dietary practices and therefore must be adapted [7]–[9] or developed [10]. Another drawback is the paucity of nutritional evidence used to construct a food-based index. In contrast, there is a large body of evidence regarding nutrient intakes (including recommended dietary intakes and lower and upper intake levels) that has not often been used to estimate the overall quality of the diet. Nutrient-based diet quality indices are robust and adaptable to different populations and countries. For example the Mean Adequacy Ratio index is used as an indicator of nutritional quality [11], [12] and the Mean Probability Adequacy index provides a composite measure of adequacy of several nutrients [13], [14]. However these indices do not take into account the upper levels of intake and therefore cannot be used to estimate the overall quality of the diet.

Lastly, it has been reported that current diet quality indices present many theoretical and methodological limitations [4]–[6], [15], including a lack of evaluation or validation. This is due partly to a lack of criterion for estimating diet quality and a lack of amenability to classical criterion validation. Nevertheless, some studies have proposed strategies to evaluate the validity of diet quality indices [10], [16] or a nutrient profile model [17] based on relevant methodologies developed in the psychometric sciences for questionnaire scales [18], [19].

The aim of this study was therefore to develop a new diet quality index based on the intake of all nutrients, using a probabilistic approach for estimating the adequacy of nutrient intake [20], and to carry out an evaluation of its validity using French and US national survey data.

## Materials and Methods

### Subjects and Data

Data used in this study came from the French Nutrition and Health Survey (Etude nationale nutrition santé - ENNS, 2006–2007) and the US National Health and Nutrition Examination Survey (NHANES, 2007–2008).

The design, methodology and results of ENNS have been described in full elsewhere [21]. Briefly, the ENNS survey was a multistage stratified descriptive cross-sectional survey undertaken on a randomly selected sample of non-institutionalized 18–74 years olds living in mainland France. Dietary data were collected using three 24-hour recalls (one of which was on the weekend) randomly selected within a 2-week period. Dietary recalls were conducted over the telephone by trained dieticians. Nutritional values for energy and nutrients came from a previously published nutrient database [22], updated to include recently marketed foods and recipes. Blood samples were collected for determination of plasma folate using competitive immunoassay with direct chemiluminescence and for determination of alpha- and beta-carotene using HPLC.

The design, methodology and results of NHANES has also been described in full elsewhere [23]. Briefly, the NHANES survey was a multistage stratified descriptive cross-sectional survey on a randomly selected sample of the civilian non-institutionalized population of the US, 20 to 80 years old. Subjects completed two 24-hour recalls, the first of which was collected in-person by trained dieticians and the second was collected over the telephone between 3 and 10 days later. Nutritional values for energy and nutrients came from the USDA’s Food and Nutrient Database for Dietary Studies 4.1 (FNDDS 4.1). Blood samples were collected for determination of plasma folate using the microbiologic assay. Carotenoid data were not collected.

In both surveys, mean individual intakes of food (in grams) and nutrients were calculated, including a weighting for the day of the week (weekday or weekend day). Nutrient intakes were expressed as absolute values and as a percentage of total energy intake, excluding energy from alcohol. In the present study, the food and drink items from ENNS (n = 1427) and NHANES (n = 7177) food composition databases were classified into thirty-seven food categories. These food categories are principally the same for the two databases however some minor discrepancies exist due to differences in data collection and coding procedures.

Of those subjects who completed the surveys (n = 3115 in ENNS and n = 5935 in NHANES), we excluded those who (i) did not provide complete dietary data (complete data was defined as three 24-hour recalls in ENNS and two 24-hour recalls in NHANES), (ii) had missing information for analysed variables or variables required for the development of the index (e.g. bodyweight), (iii) were pregnant or lactating and (iv) were identified as over- or under-reporters based on the method proposed by Black and colleagues [24]. This resulted in a final number of 1330 subjects in ENNS (43% of those who completed the survey) and 2391 subjects in NHANES (40%) available for the analysis.

### Development of a Diet Quality Index Based on the Probability of Adequate Nutrient Intake (PANDiet)

The PANDiet aims to measure the overall diet quality of an individual through the probability of having an adequate nutrient intake.

We selected 24 nutrients for inclusion in the PANDiet: protein, total carbohydrate, fibre, total fat, saturated and polyunsaturated fatty acids, cholesterol, thiamin, riboflavin, niacin, folate, vitamins A, B-6, B-12, C, D and E, calcium, magnesium, zinc, phosphorus, potassium, iron and sodium. This selection was based on the available current national nutritional recommendations for French [25]–[30] and US adults [31]–[38], and the availability of data in ENNS and NHANES food composition databases.

**Figure 1:**
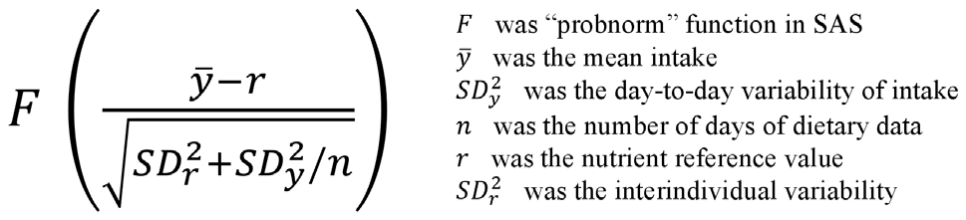
Probabilistic calculation to estimate the adequacy of the usual intake of a nutrient.

We used the probabilistic approach developed by the Institute of Medicine [20] to estimate, for each individual, if the usual intake of a nutrient was adequate. The calculation of the probability takes into account the number of days of dietary data, the mean intake and the day-to-day variability of intake, the nutrient reference value and the interindividual variability (Figure 1). Values range from 0 to 1, where 1 represents a 100% probability that the usual intake was adequate

For each nutrient, adequate intake was assumed to be the level likely to satisfy the nutrient requirements and unlikely to be excessive and elicit adverse health effects. Therefore, we assessed separately the probability that the intake was adequate inasmuch as it satisfied the requirement, on one hand, and the probability that it was not excessive, on the other hand. Consequently, the PANDiet was constructed based on two sub-scores - the Adequacy sub-score and the Moderation sub-score.

The Adequacy sub-score was calculated as the average of the probability of adequacy for items for which the usual intake should be above a reference value, multiplied by 100. According to the nutrient reference values, the probability was determined as follows:

1. For the majority of nutrients, the probability was determined from the distribution of requirements as specified by the Estimated Average Requirement (EAR) and the variability of the requirement in the specific population.
2. For some nutrients, the probability was determined from the same principle using the Adequate Intake (AI) instead of the EAR. Because interindividual variability is not specified for the AI, it was set at the same value as the variability for most nutrient requirements, 15% for France [25] and 10% for the US [20].
3. For total carbohydrate and total fat, the recommended dietary intakes are expressed as a percentage of energy intake excluding alcohol and represented by an acceptable distribution range in both French and US recommendations. The probability was calculated using the lower bounds of the acceptable distribution range. Because the use of an acceptable distribution range already accounts in part for the interindividual biological variability, no variability value was added.
4. For iron, the probability was determined using published values [31].

The Moderation sub-score was calculated as the average of the probability of adequacy for items for which the usual intake should not exceed a reference value and penalty values, multiplied by 100. According to the nutrient reference values, the probability was determined as follows:

1. For protein (upper bound), SFA, cholesterol and sodium, the probability was determined from the same principle as above, using the upper tolerable limit of intake instead of the AI. Because interindividual variability has not been specified for the upper tolerable limit, we set it at 15% for France [25] and 10% for the US [20], except for protein (upper bound) where it has been set at 0% [26].
2. For total carbohydrate and total fat, the probability of an excess in intake was calculated using upper bounds of the acceptable distribution range.

For other vitamins and minerals with available upper tolerable limits but where the risk of excessive intake is low, we used a penalty value system: a value equal to 0 was generated when the average intake of a nutrient exceeded the upper tolerable limit of intake.

The PANDiet score is the average of the Adequacy and Moderation sub-scores. In principle, the score ranges from 0 to 100; the higher the score, the better the diet quality.

**Figure 2:**
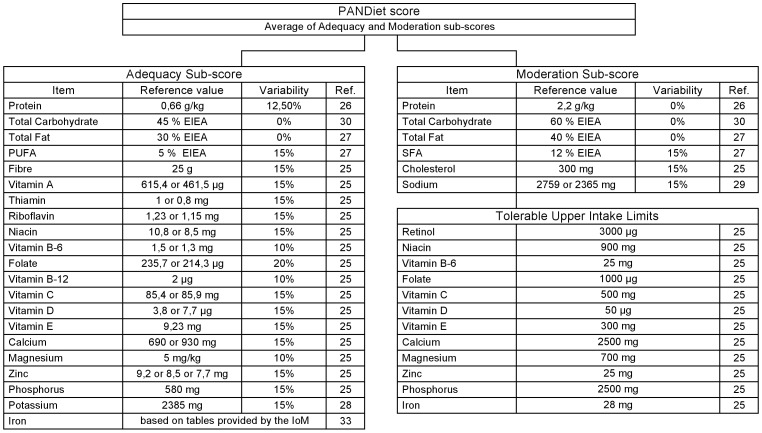
French implementation of the PANDiet: items, reference values and variabilities. The Adequacy sub-score is composed of 21 items and the Moderation sub-score is composed of 6 items plus 12 potential penalty values. EIEA, Energy Intake Excluding Alcohol.

**Figure 3:**
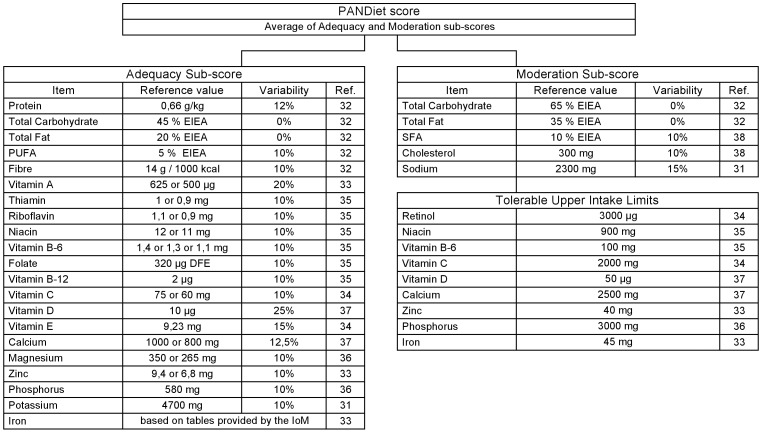
US implementation of the PANDiet: items, reference values and variabilities. The Adequacy sub-score is composed of 21 items and the Moderation sub-score is composed of 5 items plus 9 potential penalty values. EIEA, Energy Intake Excluding Alcohol.

A French implementation of the PANDiet (Figure 2) was developed based on the French nutritional recommendations for adults [25]–[27], including European Community values when specific French recommendations did not exist [28]–[30]. A US implementation of the PANDiet (Figure 3) was developed based on the US nutritional recommendations for adults [31]–[38]. Although the structure of these two implementations is almost identical, it should be noted that the differences in reference values renders cross-national comparisons of PANDiet scores meaningless.

### Evaluation of the Validity

The French and US implementations of the PANDiet were evaluated by assessing their content and construct validity.

Content validity consists of a judgment as to whether or not the index samples all the relevant or important content or domains [18], [19]. The correlations between the individual items and the PANDiet and the relationship between the PANDiet score and energy intake were investigated. The latter was checked in order to verify if a higher score would be automatically attributed to a higher energy diet.

Construct validity is an on-going process which involves three steps: 1) explicitly spelling out a set of theoretical concepts and how they are related 2) developing scales to measure these theoretical constructs and 3) testing the relationships among the constructs [18], [19]. We evaluated the construct validity of the PANDiet using different theories relating to subgroups of the population that present ‘a priori’ different diet qualities. We selected specific traits supported by literature in both France and the US:

1. We hypothesised that non-smokers have a better diet quality than smokers [39]–[41]. Accordingly, participants with a higher PANDiet score should be more likely to be non-smokers. In the present study, smokers were defined as current smokers (including heavy or occasional) and non-smokers were defined as ex- or never-smokers.
2. We hypothesised that individuals consuming a lower-energy-dense diet have a better diet quality than individuals consuming a higher-energy-dense diet [42]–[44]. Accordingly, participants with a higher PANDiet score should be more likely to have a lower-energy-dense diet. In this study, total energy density of the diet was calculated by dividing total energy intake (kcal) from food for each day by the total weight of the reported food intake (g). All beverages were excluded from this calculation based on an approach previously published [45].
3. We hypothesised that following food-based recommendations [38], [46] ensures a good nutritional quality of the diet. Accordingly, participants with a higher PANDiet score should be more likely to have food intakes in line with the international nutrition policies (e.g. more fruits and vegetables and less meat and processed meat).

In addition, given that fruit and vegetable intakes are main contributors to intakes of folate [47] and carotenoids [48], we hypothesised that higher plasma folate and carotenoids concentrations would reflect diet quality. Accordingly, participants with a higher PANDiet score should be more likely to have a higher plasma folate, alpha and beta-carotene concentrations.

### Statistical Analyses

All analyses were performed using SAS version 9.1.3 (SAS Institute). Weighting schemes proposed by ENNS and NHANES were used to account for the complex survey designs and were adapted to the population samples analyzed. To describe the distribution of the PANDiet, elemental statistics (mean, standard error of the mean and quartiles) were used. Continuous variables are presented as mean ± SEM. Because the probabilities of adequacy were not normally distributed, correlation coefficients between the PANDiet items, sub-scores, score and energy intake were assessed using Spearman’s correlations. Associations between the PANDiet (dependent variable) and sex, age, smoking status, total energy density of the diet, food intakes, plasma folate, and alpha- and beta-carotene (independent variables) were assessed in simple linear models and in a multivariate model after adjusting for age, sex and smoking status where appropriate. *P*<0.05 was considered significant.

## Results

### French Implementation of the PANDiet

The mean PANDiet score was 63.25±0.29 (range: 42.69–89.61). The PANDiet was approximately normally distributed (skewness = 0.21 and kurtosis = −0.34). The correlation with the PANDiet score was higher for the Moderation sub-score (r = 0.71) than the Adequacy sub-score (r = 0.47, Table 1). The correlations between the PANDiet score and PANDiet items were as expected, except for PUFA, zinc, vitamin A, vitamin B-12 and vitamin D (Table 1). The inter-correlations between individual items, expressed in absolute values, ranged from r = 0.00 to r = 0.84 (Table S1). The correlation with the PANDiet score was not significant for total energy intake excluding alcohol (r = −0.02, *P* = 0.50). While participants with a higher PANDiet score were more likely to be older (*P* = 0.0314), there was no significant association with sex (*P* = 0.10, Table 2).

**Table 1:** PANDiet and individual item scores shown by quartiles and Spearman correlations between the PANDiet score and individual item scores for the French sample (n = 1330).

	Q1 (n = 332)	Q2 (n = 333)	Q3 (n = 333)	Q4 (n = 332)	ρ	P-value
PANDiet score	53.56±0.23	60.08±0.12	65.71±0.14	74.20±0.28		
Moderation sub-score	43.63±0.80	51.02±0.99	59.23±1.10	71.27±0.75	0.71	<0.0001
Protein	0.95±0.01	0.96±0.02	0.99±0.00	0.99±0.01	0.07	0.0138
Total Carbohydrate	0.99±0.00	0.98±0.01	0.98±0.01	0.98±0.00	- 0.34	<0.0001
Total Fat	0.21±0.02	0.49±0.03	0.76±0.02	0.92±0.01	0.69	<0.0001
SFA	0.03±0.01	0.08±0.01	0.17±0.02	0.38±0.02	0.68	<0.0001
Cholesterol	0.22±0.02	0.30±0.02	0.38±0.03	0.67±0.02	0.45	<0.0001
Sodium	0.26±0.02	0.27±0.02	0.28±0.03	0.35±0.03	0.10	0.0002
Adequacy sub-score	63.50±0.94	69.15±0.95	72.18±1.10	77.13±0.68	0.47	<0.0001
Protein	0.98±0.00	0.97±0.01	0.99±0.00	0.99±0.00	0.12	<0.0001
Total Carbohydrate	0.11±0.02	0.32±0.03	0.53±0.03	0.76±0.03	0.61	<0.0001
Total Fat	0.98±0.01	0.94±0.01	0.89±0.02	0.77±0.02	- 0.50	<0.0001
PUFA	0.60±0.03	0.56±0.02	0.54±0.02	0.57±0.03	- 0.02	0.5429
Fibre	0.06±0.01	0.15±0.02	0.24±0.02	0.38±0.03	0.47	<0.0001
Vitamin A	0.86±0.02	0.88±0.02	0.86±0.02	0.88±0.02	0.04	0.1455
Thiamin	0.66±0.02	0.72±0.03	0.77±0.02	0.85±0.02	0.24	<0.0001
Riboflavin	0.84±0.02	0.88±0.02	0.88±0.02	0.91±0.02	0.12	<0.0001
Niacin	0.91±0.01	0.94±0.01	0.95±0.02	0.97±0.01	0.22	<0.0001
Vitamin B-6	0.62±0.03	0.72±0.03	0.78±0.03	0.86±0.02	0.35	<0.0001
Folate	0.72±0.02	0.83±0.02	0.86±0.02	0.91±0.01	0.36	<0.0001
Vitamin B-12	0.93±0.01	0.91±0.01	0.93±0.01	0.92±0.01	- 0.01	0.6719
Vitamin C	0.29±0.03	0.47±0.03	0.60±0.03	0.76±0.03	0.47	<0.0001
Vitamin D	0.11±0.02	0.12±0.02	0.13±0.02	0.10±0.02	- 0.08	0.0023
Vitamin E	0.52±0.03	0.62±0.02	0.61±0.03	0.70±0.02	0.21	<0.0001
Calcium	0.65±0.03	0.72±0.03	0.77±0.02	0.76±0.02	0.12	<0.0001
Magnesium	0.23±0.02	0.29±0.03	0.34±0.03	0.49±0.03	0.33	<0.0001
Zinc	0.78±0.02	0.78±0.02	0.80±0.02	0.78±0.02	- 0.03	0.3476
Phosphorus	0.98±0.00	0.98±0.01	0.99±0.00	0.99±0.00	0.15	<0.0001
Potassium	0.60±0.02	0.77±0.02	0.82±0.03	0.89±0.02	0.44	<0.0001
Iron	0.91±0.01	0.95±0.02	0.89±0.02	0.95±0.01	0.11	<0.0001

ENNS 2006–2007.

Values are mean ± SEM.

**Table 2:** Regression coefficients and 95% CI from linear regression analysis.

	French Sample (n = 1330)		US Sample (n = 2391)	
	β^2^ (95% CI)	P-value	β^2^ (95% CI)	P-value
Age^1^, *y*	0.04 (0.00 to 0.08)	0.0314	- 0.01 (- 0.03 to 0.01)	0.4180
Sex, Male^2^	- 0.95 (- 2.10 to 0.20)	0.1042	- 2.59 (- 3.71 to - 1.46)	0.0002
Smoking Status, Smoker^3^	- 2.25 (- 3.56 to - 0.95)	0.0007	- 3.23 (- 5.08 to - 1.38)	0.0020
Total energy density^3^, *kcal/g/d*	- 12.30 (- 14.00 to - 10.61)	<0.0001	- 4.42 (- 4.88 to - 3.96)	<0.0001
Plasma folate^4^, *ng/ml*	0.42 (0.22 to 0.62)	<0.0001	0.14 (0.09 to 0.20)	<0.0001
Alpha-carotene^4^, *µmol/l*	12.03 (7.92 to 16.14)	<0.0001	N/A	N/A
Beta-carotene^4^, *µmol/l*	3.16 (1.93 to 4.40)	<0.0001	N/A	N/A

ENNS 2006–2007 and NHANES 2007–2008.

1 Data are regression coefficients and 95% CI adjusted for sex.

2 Data are regression coefficients and 95% CI adjusted for age.

3 Data are regression coefficients and 95% CI adjusted for age and sex.

4 Data are regression coefficients and 95% CI adjusted for age, sex and smoking status.

**Figure 4:**
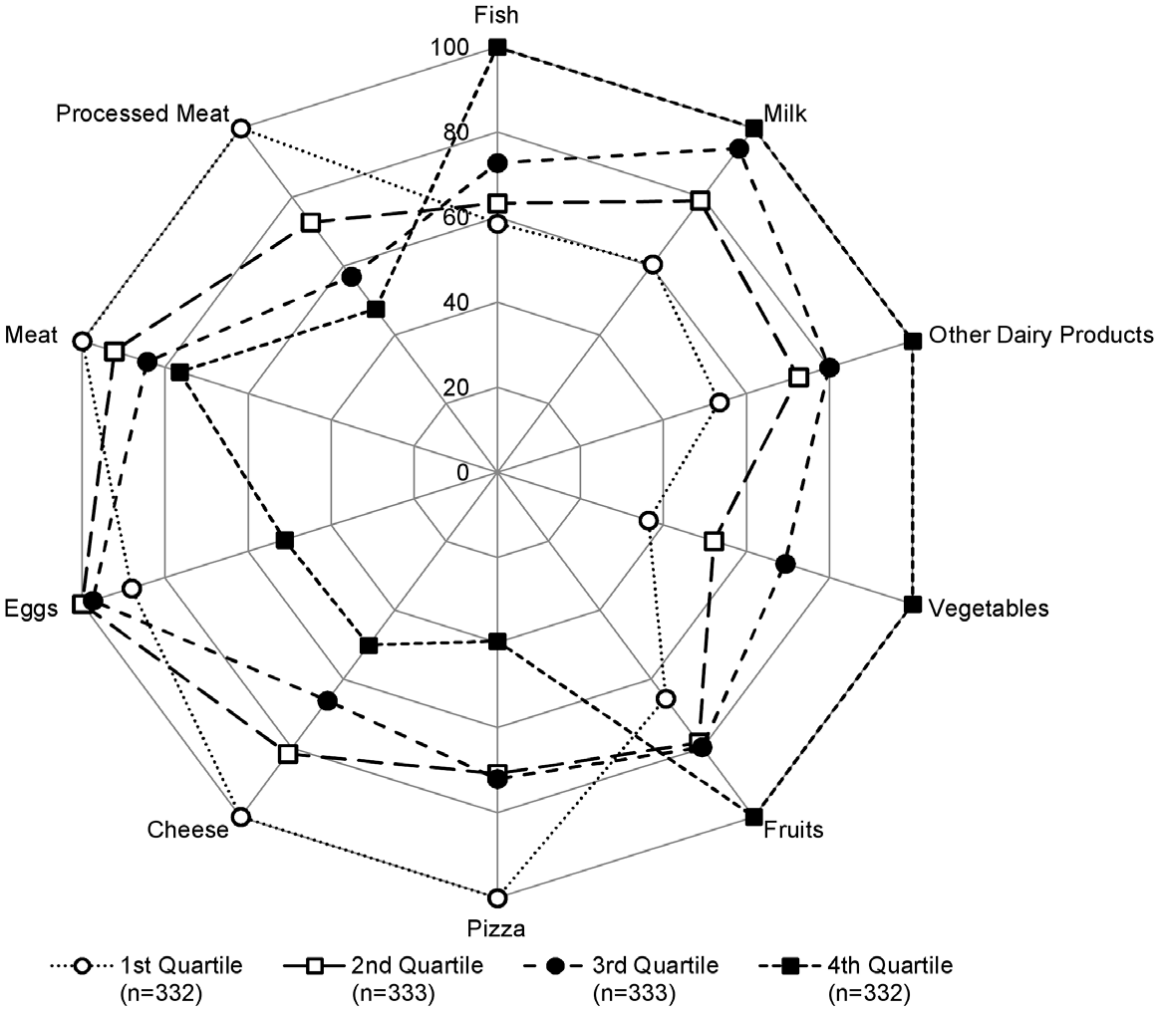
French implementation of the PANDiet and association with selected food groups. Relative mean intake of each quartile shown as a percentage compared to the highest observed mean intake across the quartiles for selected foods among the French sample. ENNS 2006–2007.

Participants with a higher PANDiet score were more likely to be non-smokers (*P* = 0.0007) and to have a lower-energy-dense diet (*P*<0.0001, Table 2). Figure 4 presents the results for the PANDiet score according to 10 food groups identified as likely to indicate diet quality, important in terms of nutrition policies and with a robust number of consumers. Full results for all food groups are shown in Table S2. Participants with a higher PANDiet score had a diet higher in the intake of milk, other dairy products (e.g. yogurt), fish, fruit and vegetables (all *P*<0.01 except for milk where *P* = 0.0237) and lower in cheese, eggs, meat, processed meat and pizza (all *P*<0.01 except for meat where *P* = 0.0131 and eggs where *P* = 0.0570). Participants with a higher PANDiet score were more likely to have higher plasma folate, alpha and beta-carotene concentrations (all *P*<0.0001, Table 2).

### US Implementation of the PANDiet

The mean PANDiet score was 58.73±0.36 (range: 34.74–89.97). The PANDiet was approximately normally distributed (skewness = 0.13 and kurtosis = −0.60). The correlation with the PANDiet score was higher for the Moderation sub-score (r = 0.82) than the Adequacy sub-score (r = 0.43, Table 3). The correlations between the PANDiet score and PANDiet items were as expected, except for PUFA, vitamin B-12 and vitamin E (Table 3). The inter-correlations between individual items, expressed in absolute values, ranged from r = 0.00 to r = 0.72 (Table S3). The correlation with the PANDiet score was significant but low for total energy intake excluding alcohol (r = −0.11, *P*<0.0001). Participants with a higher PANDiet score were more likely to be female (*P* = 0.0002) whereas there was no association with age (*P* = 0.42, Table 2).

**Table 3:** PANDiet and individual item scores shown by quartiles and Spearman correlations between the PANDiet score and individual item scores for US sample (n = 2391).

	Q1 (n = 598)	Q2 (n = 598)	Q3 (n = 598)	Q4 (n = 597)	ρ	P-value
PANDiet score	46.03±0.25	54.86±0.12	62.45±0.11	71.81±0.31		
Moderation sub-score	31.06±1.09	42.19±0.83	55.66±1.09	69.02±0.53	0.82	<0.0001
Total Carbohydrate	0.98±0.01	0.95±0.01	0.91±0.01	0.92±0.01	- 0.33	<0.0001
Total Fat	0.09±0.01	0.34±0.02	0.71±0.03	0.86±0.01	0.73	<0.0001
SFA	0.08±0.02	0.23±0.02	0.41±0.02	0.66±0.02	0.70	<0.0001
Cholesterol	0.32±0.03	0.48±0.03	0.65±0.03	0.83±0.02	0.51	<0.0001
Sodium	0.09±0.01	0.13±0.01	0.12±0.01	0.20±0.02	0.17	<0.0001
Adequacy sub-score	61.00±1.10	67.52±0.96	69.23±1.08	74.61±0.68	0.43	<0.0001
Protein	0.89±0.02	0.90±0.01	0.92±0.01	0.95±0.01	0.08	0.0002
Total Carbohydrate	0.40±0.03	0.69±0.02	0.89±0.01	0.95±0.01	0.60	<0.0001
Total Fat	0.99±0.01	0.98±0.00	0.96±0.01	0.96±0.01	- 0.32	<0.0001
PUFA	0.88±0.02	0.84±0.02	0.75±0.02	0.82±0.01	- 0.19	<0.0001
Fibre	0.01±0.00	0.05±0.01	0.08±0.01	0.18±0.02	0.46	<0.0001
Vitamin A	0.41±0.02	0.55±0.03	0.55±0.04	0.68±0.03	0.25	<0.0001
Thiamin	0.85±0.02	0.89±0.01	0.92±0.01	0.97±0.01	0.22	<0.0001
Riboflavin	0.95±0.01	0.95±0.01	0.96±0.01	0.98±0.00	0.08	0.0001
Niacin	0.93±0.01	0.96±0.01	0.96±0.01	0.97±0.01	0.13	<0.0001
Vitamin B-6	0.76±0.03	0.84±0.02	0.87±0.02	0.93±0.01	0.23	<0.0001
Folate	0.74±0.02	0.83±0.01	0.88±0.02	0.94±0.01	0.29	<0.0001
Vitamin B-12	0.89±0.01	0.90±0.01	0.88±0.02	0.92±0.01	- 0.02	0.2570
Vitamin C	0.26±0.02	0.50±0.04	0.59±0.03	0.67±0.03	0.40	<0.0001
Vitamin D	0.07±0.01	0.12±0.01	0.15±0.02	0.20±0.01	0.20	<0.0001
Vitamin E	0.15±0.02	0.23±0.02	0.16±0.01	0.25±0.02	0.05	0.0171
Calcium	0.48±0.03	0.56±0.02	0.59±0.03	0.63±0.02	0.15	<0.0001
Magnesium	0.36±0.03	0.51±0.03	0.55±0.04	0.72±0.03	0.35	<0.0001
Zinc	0.82±0.02	0.84±0.01	0.83±0.01	0.88±0.02	0.08	0.0002
Phosphorus	0.97±0.01	0.97±0.01	0.98±0.00	0.99±0.00	0.09	<0.0001
Potassium	0.05±0.01	0.11±0.01	0.08±0.01	0.10±0.01	0.19	<0.0001
Iron	0.96±0.01	0.96±0.01	0.97±0.00	0.97±0.01	0.06	0.0017

NHANES 2007–2008.

Values are mean ± SEM.

**Figure 5:**
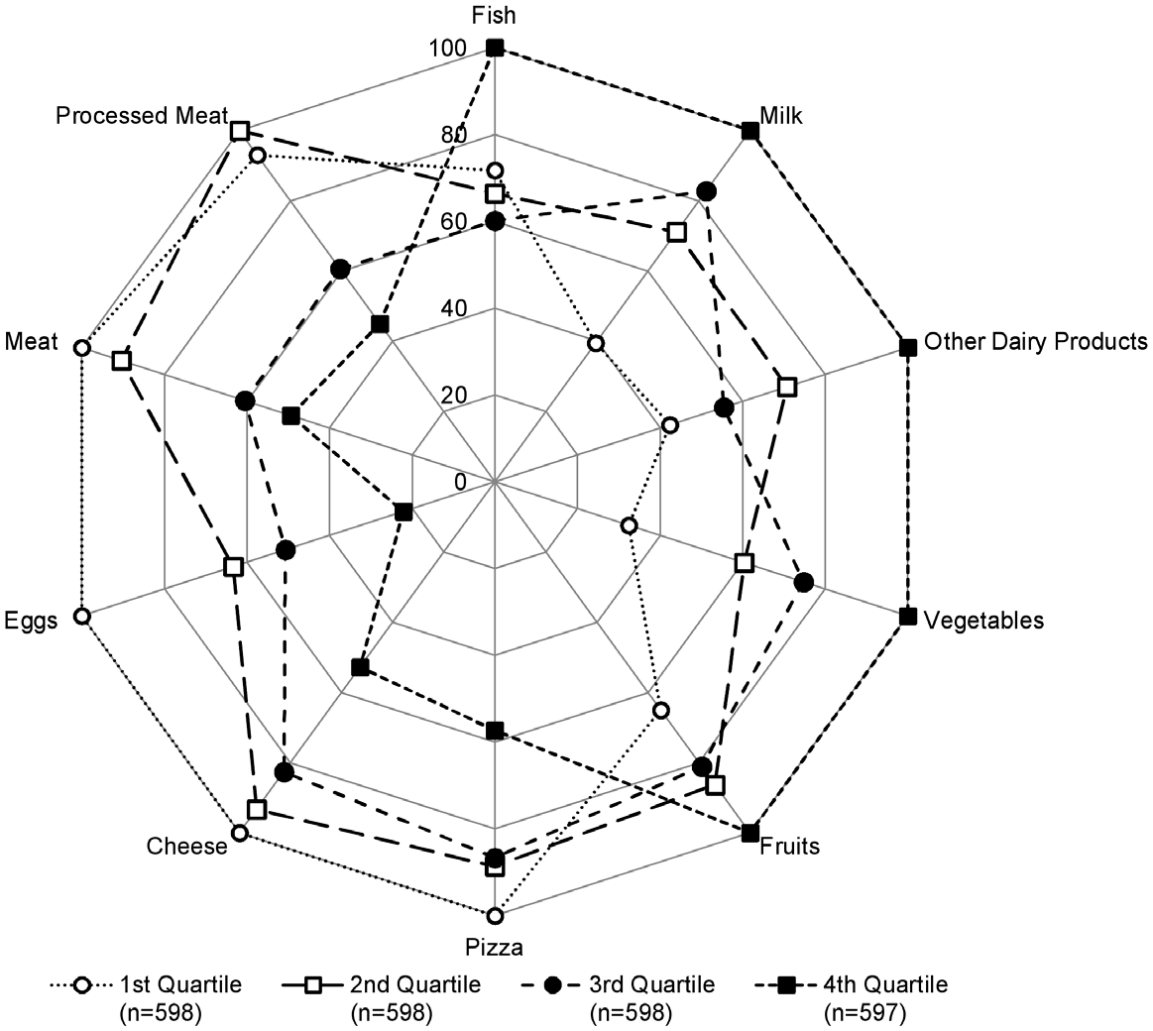
US implementation of the PANDiet and association with selected food groups. Relative mean intake of each quartile shown as a percentage compared to the highest observed mean intake across the quartiles for selected foods among US sample. NHANES 2007–2008.

Participants with a higher PANDiet score were more likely to be non-smokers (*P* = 0.0020) and to have a lower-energy-dense diet (*P*<0.0001, Table 2). As shown in Figure 5, participants with a higher PANDiet score had a diet higher in the intake of milk, other dairy products (e.g. yogurt), fish, fruit and vegetables (all *P*<0.01 except for fish where *P* = 0.0327) and lower in intakes of cheese, eggs, meat, processed meat and pizza (all *P*<0.01). Full results are shown in Table S2. Participants with a higher PANDiet score were more likely to have a higher plasma folate concentration (*P*<0.0001, Table 2).

## Discussion

The present study describes the development of a new diet quality index, the PANDiet. This index provides a measure of overall diet quality and each PANDiet item assesses the probability of adequate nutrient intake according to a specific nutritional reference. We report the strategy used to evaluate the validity of this index, and the ensuing validity elements based on the application of the PANDiet to data from two different populations.

The correlation between the PANDiet score and PANDiet items reflect the contribution of the variation of each item to the variation of the PANDiet score. In both implementations, we found that the items related to total carbohydrates (lower bound), total fat (upper bound), SFA, fibre and vitamin C had the most important influence on the PANDiet score and thus, satisfying the recommendations for these nutrients were the most important factors in discriminating the diet quality of the population samples analyzed. Conversely, low correlations reflected that some nutritional recommendations were not discriminating factors and the related items did not influence the PANDiet score (e.g. vitamin D). Nevertheless, such items still provide important information and need to be taken into account in an overall assessment of diet quality.

Recent publications have emphasized that diet quality indices developed to date present several unresolved methodological issues that may reduce their diagnostic capacity [4]–[6], [15]. One issue concerns the existence of high inter-correlations between index items that may lead to an undesirable over-contribution of some items to the score. The inter-correlations between items of the PANDiet reflect the complexity of the diet and interactions between dietary and nutrient intakes. These inter-correlations do not point to a problem of assessing similar aspects of the diet with different items. Because of the lack of a science-based rationale to develop a weighting system for the nutrients, we used an equal weighting for nutrients within each sub-score of the PANDiet. It should be noted that using two sub-scores and averaging their scores to provide the final PANDiet score designates a higher weight to the items of the Moderation sub-score than to the items of the Adequacy sub-score since the former includes fewer items than the latter.

Like very few other diet quality indices [10], [16], the validity of the PANDiet was evaluated through a strategy based on methodologies developed in the psychometric sciences [18], [19]. The PANDiet passed the different tests of validity that were based on factors considered to be associated with diet quality from the literature in both France and the US. We have shown that the PANDiet was in line with published findings that consistently indicate smokers have higher intakes of total fat and SFA, and lower intakes of folate, vitamin C and fibre compared with non-smokers [39]–[41]. This ability to detect differences in the quality of the diet of smokers and non-smokers has also been reported for several other diet quality indices [7], [10], [16], [49]–[52]. We have also shown that the PANDiet assesses nutrient adequacy independently of energy intake, as demonstrated by the absence of a correlation in the French sample and a very low correlation in the US sample between the PANDiet score and total energy intake. Furthermore, the significant negative association with energy density indicates that a higher PANDiet score reflects diets that are nutrient but not energy dense. Low or insignificant correlations between the total score and total energy intake have been reported for several diet quality indices [10], [16], [50] but the association with energy density has been rarely investigated [53]. Lastly, we have shown that the PANDiet assesses diet quality in terms of relative food consumption. The variation in the intake of ten food groups presented according to the PANDiet score are in line with the international nutrition policies [38], [46] and diet modelling based on current nutritional recommendations [54], [55]: lowering the intakes of several animal products (e.g. meat and processed meat), increasing that of fruits, vegetables and fish and equilibrating the intake of items within the dairy product category (lowering the intake of higher fat cheeses in favour of lower fat milks or yogurts).

Unfortunately some nutrients could not be included in the index despite nutritional recommendations existing (e.g. added sugars) due to a lack of data in the food composition databases. Similarly, items estimating the probability of an adequate intake of simple and complex sugars could not be included due to a lack of specific nutritional recommendations. Nevertheless, when such recommendations are developed or updated or nutrient composition information is available, it will be possible to include new items in the PANDiet and confirm the validity of the updated index. Lastly, it should be noted that the restricted samples on which these analyses were undertaken could limit the representativeness of the findings and the generalizability of the results. The use of relevant weighting schemes has limited this potential bias.

The majority of other published diet quality indices rely on food-based dietary guidelines, which simplifies the selection of the items in the index, the scoring system and the weighting. Since this approach does not require a translation of food intakes into nutrient intakes, it therefore enables the application to shorter or less detailed methods of dietary assessment, which are often used in field research. In addition, this approach indirectly assesses intakes of nutrient and non-nutrient components in food. However, food-based dietary guidelines are drawn from a mix of different nutrition knowledge: some recommendations are based on epidemiological data that have ascertained a relationship with a health-related outcome (e.g. intake of fruits and vegetables), other food intake recommendations arise indirectly from a recommendation in nutrient intake (e.g. intake of dairy products in relation to the requirement for calcium), or, even more indirectly to the place left for some food categories once the frequency or amount of others have been defined. Therefore, food-based dietary guidelines account for nutrient intake recommendations only very indirectly. Accordingly, scoring using food-based dietary guidelines does not use the precise information of food and diet quality at the individual level. One example of the mismatch between food-based dietary guidelines and nutrient adequacy is that adherence to food patterns built from food-based dietary guidelines does not always ensure adequate intake of several nutrients, such as vitamin E or potassium [56]. The large heterogeneity commonly found within food groups in terms of nutrient density tends to reduce the sensitivity of the index. Furthermore, food-based dietary guideline indices have to be adapted [7]–[9] in order to be used in countries with different dietary practices. Indeed, nutrient requirements can be covered in many different ways, which explain why considering the nutrient level can assess more accurately the quality of the diet at the individual level. In the PANDiet, which is a diet quality index based only on nutrients, this accuracy is strengthened by the use of the probabilistic calculation of nutrient adequacy. The PANDiet accounts for the precision of the estimation of usual intakes of nutrients from dietary surveys, and utilizes all current knowledge based on nutrient intakes (including EAR, AI, and tolerable upper limit of intake). Finally, the PANDiet offers a complete diet quality index relevant at the nutrient level. For studying the diet quality of populations, the PANDiet appears complementary to indices relying on food based patterns (e.g. Mediterranean diets). At the individual level, the PANDiet offers an accurate index to qualify the diet quality that could be used for individual diagnosis and follow-up in the framework of tailored dietary advice.

In conclusion, there is strong evidence suggesting that the PANDiet is a useful tool to assess diet quality at the population level. Although this study concerns the French and US general adult populations, the PANDiet could be applied to other countries or specific populations, where relevant nutritional recommendations and nationally or specific population representative dietary data are available. Further validation of the PANDiet would require the examination of the relationship between the PANDiet score and a large set of biochemical and clinical indicators of nutritional status. The PANDiet stands as a useful tool to explore how diet quality, as captured by this nutrient-based index, relates to risk of morbidity and mortality using longitudinal surveys.

## Supporting Information

Table S1 **Spearman correlations between PANDiet items scores among French sample (n = 1330).** ENNS 2006–2007.
(XLS)

Table S2 **Regression coefficients and 95% CI from linear regression analysis of the PANDiet score for intakes of the thirty-seven food categories adjusted for age and sex.** ENNS 2006–2007 and NHANES 2007–2008^1^
(DOCX)

Table S3 **Spearman correlations between PANDiet items scores among US sample (n = 2391).** NHANES 2007–2008.
(XLS)

## References

[1] Hu FB (2002) Dietary pattern analysis: a new direction in nutritional epidemiology. Curr Opin Lipidol 13: 3–9. 11790957 10.1097/00041433-200202000-00002

[2] Kant AK (2004) Dietary patterns and health outcomes. J Am Diet Assoc 104: 615–635. 15054348 10.1016/j.jada.2004.01.010

[3] Newby PK, Tucker KL (2004) Empirically derived eating patterns using factor or cluster analysis: a review. Nutr Rev 62: 177–203. 15212319 10.1301/nr.2004.may.177-203

[4] Waijers PM, Feskens EJ, Ocke MC (2007) A critical review of predefined diet quality scores. Br J Nutr 97: 219–231. 17298689 10.1017/S0007114507250421

[5] Kourlaba G, Panagiotakos DB (2009) Dietary quality indices and human health: a review. Maturitas 62: 1–8. 19128905 10.1016/j.maturitas.2008.11.021

[6] Wirt A, Collins CE (2009) Diet quality–what is it and does it matter? Public Health Nutr 12: 2473–2492. 19335941 10.1017/S136898000900531X

[7] McNaughton SA, Ball K, Crawford D, Mishra GD (2008) An index of diet and eating patterns is a valid measure of diet quality in an Australian population. J Nutr 138: 86–93. 18156409 10.1093/jn/138.1.86

[8] Taechangam S, Pinitchun U, Pachotikarn C (2008) Development of nutrition education tool: healthy eating index in Thailand. Asia Pac J Clin Nutr 17 Suppl 1: 365–367. 18296380

[9] Shatenstein B, Nadon S, Godin C, Ferland G (2005) Diet quality of Montreal-area adults needs improvement: estimates from a self-administered food frequency questionnaire furnishing a dietary indicator score. J Am Diet Assoc 105: 1251–1260. 16182642 10.1016/j.jada.2005.05.008

[10] Drake I, Gullberg B, Ericson U, Sonestedt E, Nilsson J, et al (2011) Development of a diet quality index assessing adherence to the Swedish nutrition recommendations and dietary guidelines in the Malmo Diet and Cancer cohort. Public Health Nutr 7: 1–11. 10.1017/S1368980010003848 21299917

[11] Madden JP, Yoder MD (1972) Program evaluation: food stamps and commodity distribution in rural areas of central Pennsylvania. Penn Agr Exp Sta Bull 78: 1–119.

[12] Maillot M, Darmon N, Vieux F, Drewnowski A (2007) Low energy density and high nutritional quality are each associated with higher diet costs in French adults. Am J Clin Nutr 86: 690–696. 17823434 10.1093/ajcn/86.3.690

[13] Foote JA, Murphy SP, Wilkens LR, Basiotis PP, Carlson A (2004) Dietary variety increases the probability of nutrient adequacy among adults. J Nutr 134: 1779–1785. 15226469 10.1093/jn/134.7.1779

[14] Kennedy G, Fanou-Fogny N, Seghieri C, Arimond M, Koreissi Y, et al (2010) Food groups associated with a composite measure of probability of adequate intake of 11 micronutrients in the diets of women in urban Mali. J Nutr 140: 2070S–2078S. 20881080 10.3945/jn.110.123612

[15] Panagiotakos D (2009) Health measurement scales: methodological issues. Open Cardiovasc Med J 3: 160–165. 20054421 10.2174/1874192400903010160 PMC2801875

[16] Guenther PM, Reedy J, Krebs-Smith SM, Reeve BB (2008) Evaluation of the Healthy Eating Index-2005. J Am Diet Assoc 108: 1854–1864. 18954575 10.1016/j.jada.2008.08.011

[17] Arambepola C, Scarborough P, Rayner M (2008) Validating a nutrient profile model. Public Health Nutr 11: 371–378. 17605841 10.1017/S1368980007000377

[18] Streiner D, Norman G (2008) Health Measurement Scales: A Practical Guide to their Development and Use. Oxfrod: Oxford University Press.

[19] Bland JM, Altman DG (2002) Statistics Notes: Validating scales and indexes. BMJ 324: 606–607. 11884331 10.1136/bmj.324.7337.606 PMC1122519

[20] Institute of Medicine Food and Nutrition Board (2000) Dietary Reference Intakes. Applications in Dietary Assessment. Washington, DC: National Academy Press.

[21] Castetbon K, Vernay M, Malon A, Salanave B, Deschamps V, et al (2009) Dietary intake, physical activity and nutritional status in adults: the French nutrition and health survey (ENNS, 2006–2007). Br J Nutr 102: 733–743. 19250574 10.1017/S0007114509274745

[22] Hercberg S (2005) Table de composition des aliments - SU.VI.MAX. Paris: Economica.

[23] National Center for Health Statistics (2011) 2007–2008 National Health and Nutrition Examination Survey (NHANES). Available: http://www.cdc.gov/nchs/nhanes/nhanes2007-2008/nhanes07_08.htm. Accessed 2012 Jan18.

[24] Black AE (2000) Critical evaluation of energy intake using the Goldberg cut-off for energy intake:basal metabolic rate. A practical guide to its calculation, use and limitations. Int J Obes Relat Metab Disord 24: 1119–1130. 11033980 10.1038/sj.ijo.0801376

[25] Martin A (2001) Apports nutritionnels conseillés pour la population Française. Paris, France.

[26] AFSSA (2007) Apport en protéines : consommation, qualité,besoins et recommandations. Available: http://www.anses.fr/Documents/NUT-Ra-Proteines.pdf. Accessed 2012 January 18.

[27] AFSSA (2010) Avis de l’Agence française de sécurité sanitaire des aliments relatif à l’actualisation des apports nutritionnels conseillés pour les acides gras. Available: http://www.anses.fr/Documents/NUT2006sa0359.pdf. Accessed 2012 January 18.

[28] Commission of the European Communities (1993) Nutrient and energy intakes for the European Community. Available: http://ec.europa.eu/food/fs/sc/scf/out89.pdf. Accessed 2012 January 18.

[29] Pietinen P, Valsta LM, Hirvonen T, Sinkko H (2008) Labelling the salt content in foods: a useful tool in reducing sodium intake in Finland. Public Health Nutr 11: 335–340. 17605838 10.1017/S1368980007000249

[30] EFSA Panel on Dietetic Products NaA (2010) Scientific Opinion on Dietary Reference Values for carbohydrates and dietary fibre. Available: http://www.efsa.europa.eu/fr/efsajournal/doc/1462.pdf. Accessed 2012 Jan 18.

[31] Institute of Medicine Food and Nutrition Board (2004) Dietary Reference Intakes: Water, Potassium, Sodium, Chloride, and Sulfate. Washington, DC: National Academy Press.

[32] Institute of Medicine Food and Nutrition Board (2002) Dietary Reference Intakes for Energy, Carbohydrate, Fiber, Fat, Fatty Acids, Cholesterol, Protein, and Amino Acids. Washington, DC: National Academy Press. 10.1016/s0002-8223(02)90346-9 12449285

[33] Institute of Medicine Food and Nutrition Board (2001) Dietary Reference Intakes for Vitamin A, Vitamin K, Arsenic, Boron, Chromium, Copper, Iodine, Iron, Manganese, Molybdenum, Nickel, Silicon, Vanadium, and Zinc. Washington, DC: National Academy Press. 25057538

[34] Institute of Medicine Food and Nutrition Board (2000) Dietary Reference Intakes for Vitamin C, Vitamin E, Selenium, and Carotenoids. Washington, DC: National Academy Press. 25077263

[35] Institute of Medicine Food and Nutrition Board (2000) Dietary Reference Intakes for Thiamin, Riboflavin, Niacin, Vitamin B6, Folate, Vitamin B12, Pantothenic Acid, Biotin, and Choline. Washington, DC: National Academy Press. 23193625

[36] Institute of Medicine Food and Nutrition Board (1997) Dietary Reference Intakes for Calcium, Phosphorus, Magnesium, Vitamin D, and Fluoride. Washington, DC: National Academy Press. 23115811

[37] Institute of Medicine Food and Nutrition Board (2010) Dietary Reference Intakes for Vitamin D and Calcium 2010 Brief Report. Washington, DC: National Academy Press.

[38] U.S.Department of Agriculture and U.S.Department of Health and Human Services (2010) Dietary Guidelines for Americans, 2010. Washington, DC: U.S. Government Printing Office.

[39] Dallongeville J, Marecaux N, Fruchart JC, Amouyel P (1998) Cigarette smoking is associated with unhealthy patterns of nutrient intake: a meta-analysis. J Nutr 128: 1450–1457. 9732304 10.1093/jn/128.9.1450

[40] Palaniappan U, Jacobs SL, O’Loughlin J, Gray-Donald K (2001) Fruit and vegetable consumption is lower and saturated fat intake is higher among Canadians reporting smoking. J Nutr 131: 1952–1958. 11435513 10.1093/jn/131.7.1952

[41] Subar AF, Harlan LC, Mattson ME (1990) Food and nutrient intake differences between smokers and non-smokers in the US. Am J Public Health 80: 1323–1329. 2240298 10.2105/ajph.80.11.1323 PMC1404910

[42] Darmon N, Briend A, Drewnowski A (2004) Energy-dense diets are associated with lower diet costs: a community study of French adults. Public Health Nutr 7: 21–27. 14972068 10.1079/phn2003512

[43] Ledikwe JH, Blanck HM, Khan LK, Serdula MK, Seymour JD, et al (2006) Low-energy-density diets are associated with high diet quality in adults in the United States. J Am Diet Assoc 106: 1172–1180. 16863711 10.1016/j.jada.2006.05.013

[44] Schroder H, Vila J, Marrugat J, Covas MI (2008) Low energy density diets are associated with favorable nutrient intake profile and adequacy in free-living elderly men and women. J Nutr 138: 1476–1481. 18641194 10.1093/jn/138.8.1476

[45] Ledikwe JH, Blanck HM, Khan LK, Serdula MK, Seymour JD, et al (2005) Dietary energy density determined by eight calculation methods in a nationally representative United States population. J Nutr 135: 273–278. 15671225 10.1093/jn/135.2.273

[46] Hercberg S, Chat-Yung S, Chauliac M (2008) The French National Nutrition and Health Program: 2001–2006–2010. Int J Public Health 5: 68–77. 10.1007/s00038-008-7016-2 18681335

[47] Park JY, Nicolas G, Freisling H, Biessy C, Scalbert A, et al (2011) Comparison of standardised dietary folate intake across ten countries participating in the European Prospective Investigation into Cancer and Nutrition. Br J Nutr 1: 1–18. 10.1017/S0007114511005733 22040523

[48] Murphy MM, Barraj LM, Herman D, Bi X, Cheatham R, et al (2011) Phytonutrient Intake by Adults in the United States in Relation to Fruit and Vegetable Consumption. J Am Diet Assoc 112: 222–229. 10.1016/j.jada.2011.08.044 22741166

[49] Fung TT, Chiuve SE, McCullough ML, Rexrode KM, Logroscino G, et al (2008) Adherence to a DASH-style diet and risk of coronary heart disease and stroke in women. Arch Intern Med 168: 713–720. 18413553 10.1001/archinte.168.7.713

[50] Fogli-Cawley JJ, Dwyer JT, Saltzman E, McCullough ML, Troy LM, et al (2006) The 2005 Dietary Guidelines for Americans Adherence Index: development and application. J Nutr 136: 2908–2915. 17056821 10.1093/jn/136.11.2908

[51] McCullough ML, Feskanich D, Stampfer MJ, Giovannucci EL, Rimm EB, et al (2002) Diet quality and major chronic disease risk in men and women: moving toward improved dietary guidance. Am J Clin Nutr 76: 1261–1271. 12450892 10.1093/ajcn/76.6.1261

[52] Estaquio C, Kesse-Guyot E, Deschamps V, Bertrais S, Dauchet L, et al (2009) Adherence to the French Programme National Nutrition Sante Guideline Score is associated with better nutrient intake and nutritional status. J Am Diet Assoc 109: 1031–1041. 19465185 10.1016/j.jada.2009.03.012

[53] Golley RK, Hendrie GA, McNaughton SA (2011) Scores on the dietary guideline index for children and adolescents are associated with nutrient intake and socio-economic position but not adiposity. J Nutr 141: 1340–1347. 21613454 10.3945/jn.110.136879

[54] Masset G, Monsivais P, Maillot M, Darmon N, Drewnowski A (2009) Diet optimization methods can help translate dietary guidelines into a cancer prevention food plan. J Nutr 139: 1541–1548. 19535422 10.3945/jn.109.104398

[55] Maillot M, Vieux F, Amiot MJ, Darmon N (2010) Individual diet modeling translates nutrient recommendations into realistic and individual-specific food choices. Am J Clin Nutr 91: 421–430. 19939986 10.3945/ajcn.2009.28426

[56] Guenther PM, Reedy J, Krebs-Smith SM (2008) Development of the Healthy Eating Index-2005. J Am Diet Assoc 108: 1896–1901. 18954580 10.1016/j.jada.2008.08.016

