# Supplementary material for: Evaluation of a Diet Quality Index Based on the Probability of Adequate Nutrient Intake (PANDiet) Using National French and US Dietary Surveys
Source: PLoS One. 2012 Aug 3;7(8):e42155. doi: 10.1371/journal.pone.0042155 (PMC3411671; doi:10.1371/journal.pone.0042155)
Supplement: Table S2 — Regression coefficients and 95% CI from linear regression analysis of the PANDiet score for intakes of the thirty-seven food categories adjusted for age and sex. ENNS 2006–2007 and NHANES 2007–20081 (DOCX) [file pone.0042155.s002.docx]

**Table S2** Regression coefficients and 95% CI from linear regression analysis of the PANDiet score for intakes of the thirty-seven food categories adjusted for age and sex. ENNS 2006-2007 and NHANES 2007-2008^1^

|  | French Sample (n=1330) | | US Sample (n=2391) | |
| --- | --- | --- | --- | --- |
|  | β² (95% CI) | P-value | β² (95% CI) | P-value |
| Breads | 1.59 (0.70 to 2.47) | 0.0004 | 0.16 (‐ 1.19 to 1.51) | 0.8059 |
| Breakfast Cereals | 13.48 (9.61 to 17.34) | <0.0001 | 11.68 (10.17 to 13.19) | <0.0001 |
| Cereals | 0.10 (‐ 0.09 to 1.10) | 0.8445 | 2.10 (1.51 to 2.69) | <0.0001 |
| Pastries | ‐ 3.80 (‐ 5.49 to ‐ 2.12) | <0.0001 | ‐ 3.26 (‐ 6.81 to 0.28) | 0.0686 |
| Biscuits | 0.90 (‐ 2.65 to 4.45) | 0.6178 | 1.87 (1.10 to 2.64) | 0.0001 |
| Cakes | ‐ 0.52 (‐ 1.36 to 0.33) | 0.2321 | - 0.46 (‐1.42 to 0.50) | 0.3225 |
| Milk | 0.74 (0.10 to 1.37) | 0.0237 | 0.97 (0.73 to 1.21) | <0.0001 |
| Other Dairy Products | 2.33 (1.62 to 3.03) | <0.0001 | 2.36 (1.04 to 3.68) | 0.0016 |
| Cream Desserts | - 0.23 (‐ 1.28 to 0.83) | 0.6743 | 0.86 (‐ 0.68 to 2.41) | 0.2530 |
| Cheese | ‐ 6.20 (‐ 7.62 to ‐ 4.77) | <0.0001 | ‐ 4.57 (‐6.21 to ‐2.93) | <0.0001 |
| Eggs | ‐ 2.67 (‐ 5.43 to 0.08) | 0.0570 | ‐ 5.54 (‐ 6.91 to ‐4.17) | <0.0001 |
| Butter | ‐ 14.95 (‐ 21.05 to ‐ 8.85) | <0.0001 | ‐ 20.28 (‐ 38.08 to ‐ 1.47) | 0.0362 |
| Oils | 10.16 (‐ 0.62 to 20.94) | 0.0647 | 0.58 (‐ 15.01 to 16.17) | 0.9382 |
| Margarine | 9.54 (- 0.25 to 19.34) | 0.0562 | ‐ 5.15 (‐ 18.38 to 8.07) | 0.4212 |
| Meat | ‐ 1.33 (‐ 2.39 to - 0.28) | 0.0131 | ‐ 2.46 (‐ 3.19 to ‐ 1.73) | <0.0001 |
| Poultry | - 0.17 (‐ 1.21 to 0.88) | 0.7564 | ‐ 1.74 (‐ 3.00 to - 0.48) | 0.0098 |
| Offal | ‐ 2.43 (‐ 6.26 to 1.40) | 0.2143 | ‐ 3.76 (‐ 6.86 to ‐ 0.65) | 0.0208 |
| Processed Meat | ‐ 5.39 (‐ 6.61 to ‐ 4.17) | <0.0001 | ‐ 3.95 (‐ 5.48 to ‐ 2.42) | 0.0001 |
| Fish | 2.50 (1.01 to 3.98) | 0.0010 | 1.69 (0.16 to 3.21) | 0.0327 |
| Shellfish | ‐ 2.22 (‐ 4.09 to - 0.34) | 0.0204 | ‐ 1.49 (‐3.08 to 0.10) | 0.0646 |
| Vegetables | 2.26 (1.90 to 2.64) | <0.0001 | 2.47 (1.85 to 3.08) | <0.0001 |
| Potatoes | 1.22 (0.16 to 2.28) | 0.0239 | ‐ 0.93 (‐ 1.84 to ‐ 0.01) | 0.0486 |
| Beans and pulses | 4.16 (2.46 to 5.86) | <0.0001 | 1.66 (0.71 to 2.61) | 0.0019 |
| Fruits | 1.83 (1.28 to 2.39) | <0.0001 | 1.47 (0.96 to 1.98) | <0.0001 |
| Processed Fruits | 2.03 (0.29 to 3.76) | 0.0222 | 4.30 (1.42 to 7.17) | 0.0059 |
| Dried fruits & Seeds | 3.20 (‐ 1.23 to 7.64) | 0.1567 | 4.98 (1.30 to 8.66) | 0.0112 |
| Ice cream | ‐ 1.27 (‐ 3.52 to 0.98) | 0.2674 | ‐ 1.96 (‐ 3.02 to ‐ 0.89) | 0.0013 |
| Chocolate | 1.52 (‐ 2.16 to 5.20) | 0.4174 | ‐ 1.35 (‐ 5.44 to 2.74) | 0.4948 |
| Sugar to Sweets to Jams & Honey | 3.86 (1.29 to 6.42) | 0.0033 | ‐ 0.18 (‐ 2.09 to 1.73) | 0.8413 |
| Water | 0.07 (- 0.20 to 0.16) | 0.1256 | 0.08 (‐ 0.01 to 0.16) | 0.0681 |
| Soft drinks including fruit juices | 0.23 (- 0.05 to 0.51) | 0.1109 | ‐ 0.08 (‐ 0.20 to 0.04) | 0.1599 |
| Alcoholic Beverages | 0.21 (‐ 0.55 to 0.13) | 0.2275 | ‐ 0.12 (‐ 0.25 to 0.01) | 0.0724 |
| Hot Beverages | - 0.02 (- 0.20 to 0.16) | 0.7984 | ‐ 0.10 (‐ 0.22 to 0.03) | 0.1159 |
| Pizza | ‐ 1.83 (‐ 3.10 to ‐ 0.55) | 0.0051 | ‐ 0.78 (‐ 1.27 to - 0.28) | 0.0043 |
| Sandwiches & Hamburgers | 0.20 (- 1.29 to 1.69) | 0.7896 | ‐ 1.60 (‐ 2.62 to ‐ 0.58) | 0.0042 |
| Soups | 0.89 (0.46 to 1.32) | <0.0001 | 0.73 (0.37 to 1.09) | 0.0006 |
| Complex and ready-prepared dishes | ‐ 0.59 (‐ 1.24 to 0.07) | 0.0804 | 0.21 (‐ 0.05 to 0.47) | 0.1002 |

^1^ Intakes of food categories are expressed in 100g/day
